# Differentiation of Sinoatrial-like Cardiomyocytes as a Biological Pacemaker Model

**DOI:** 10.3390/ijms25179155

**Published:** 2024-08-23

**Authors:** Yvonne Sleiman, Jean-Baptiste Reisqs, Mohamed Boutjdir

**Affiliations:** 1 Cardiovascular Research Program, VA New York Harbor Healthcare System, New York, NY 11209, USA; yvonne_sleiman@hotmail.com (Y.S.); jeanbaptiste.reisqs@gmail.com (J.-B.R.); 2 Department of Medicine, Cell Biology and Pharmacology, State University of New York Downstate Health Sciences University, New York, NY 11203, USA; 3 Department of Medicine, New York University Grossman School of Medicine, New York, NY 10016, USA

**Keywords:** SAN-like cardiomyocytes, biological pacemakers, arrythmias-related pacemaker dysfunction, hESCs and hiPSCs-directed pacemaker differentiation protocols

## Abstract

Human induced pluripotent stem cell-derived cardiomyocytes (hiPSC-CMs) are widely used for disease modeling and pharmacological screening. However, their application has mainly focused on inherited cardiopathies affecting ventricular cardiomyocytes, leading to extensive knowledge on generating ventricular-like hiPSC-CMs. Electronic pacemakers, despite their utility, have significant disadvantages, including lack of hormonal responsiveness, infection risk, limited battery life, and inability to adapt to changes in heart size. Therefore, developing an in vitro multiscale model of the human sinoatrial node (SAN) pacemaker using hiPSC-CM and SAN-like cardiomyocyte differentiation protocols is essential. This would enhance the understanding of SAN-related pathologies and support targeted therapies. Generating SAN-like cardiomyocytes offers the potential for biological pacemakers and specialized conduction tissues, promising significant benefits for patients with conduction system defects. This review focuses on arrythmias related to pacemaker dysfunction, examining protocols’ advantages and drawbacks for generating SAN-like cardiomyocytes from hESCs/hiPSCs, and discussing therapeutic approaches involving their engraftment in animal models.

## 1. Introduction

Cardiovascular disease (CVD) is the leading cause of death worldwide and accounts for 33% of all deaths [1]. Despite significant progress, CVD deaths’ number has increased by approximately 61.2% from 1990 to 2021 [2]. They manifest as loss of heart function, including abnormal blood pumping, impaired mechanical contraction and electrical disturbances, hypertension, myocardial infarction, heart failure, and arrhythmias [3]. Transgenic animal models, in particular genetically modified mice carrying causative variants, have been widely used over the past two decades to study the molecular basis of CVD [4,5,6]. Although these models recapitulate some of the features of the human cardiac phenotype and provide both a structural and physiological presentation of the disease, considerable interspecies differences reside between animal model cardiomyocytes and human cardiomyocytes [7]. This highlights an important challenge in these studies, the transposition of the mechanisms identified in mice to humans [7]. The electrophysiological properties of the mouse heart are very different from those of humans both in terms of action potential and ECG morphology. For example, the resting heart rate of mice is approximately ten times faster than that of humans, and as a result, mouse cardiomyocytes show considerable differences from the human cardiomyocytes, including myofilament composition, energy metabolism, calcium (Ca^2+^) homeostasis, ion channel expression, and electrical properties [8].

**Figure 1:**
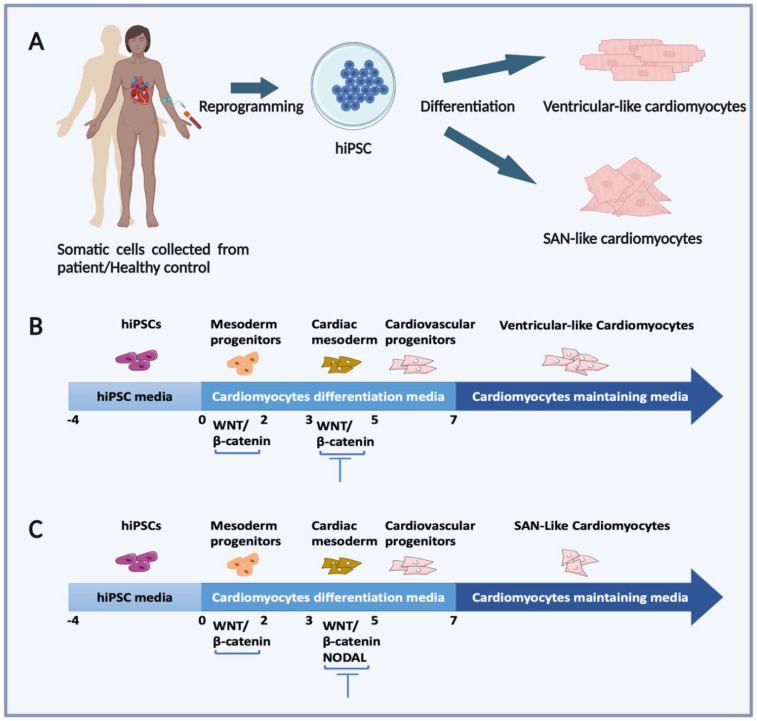
Illustration depicting the generation of patient-specific hiPSC-derived cardiomyocytes. (**A**) hiPSCs are reprogrammed from somatic cells such as blood or fibroblasts. These hiPSCs have the potential to differentiate into various cell types, including ventricular-like and sinoatrial node (SAN)-like cardiomyocytes. (**B**) Illustration of a general protocol to obtain ventricular-like cardiomyocytes. hiPSCs are maintained until confluence in an hiPSC-maintaining medium. Cardiac differentiation is initiated on day 0 by activating the WNT/β-catenin signaling pathway, leading to mesoderm formation. Cardiac progenitors are induced by inactivating the WNT/β-catenin pathway signaling pathway. Cardiovascular progenitors are typically induced by day 5 of differentiation. (**C**) Illustration of a general protocol for obtaining SAN-like cardiomyocytes. SAN-like cardiomyocytes are generated by inhibiting the NODAL pathway on day 3 of differentiation. More complex protocols are detailed in Section 3.

The human induced pluripotent stem cells (hiPSCs) emerged as an alternative model to study human diseases. hiPSCs are self-renewable cells and have the potential to theoretically differentiate into many types of somatic cells [9]. hiPSCs offer an excellent tool to decipher the mechanisms responsible for CVD and to test new therapeutic compounds in a human-derived cell model without ethical concerns [7,10,11,12,13,14]. Over the past decade, the hiPSC-derived cardiomyocytes (hiPSC-CMs) model was used to characterize these cells and model hereditary cardiac pathologies (Figure 1A,B) [15,16,17,18,19,20,21,22,23,24]. However, the hiPSC-CMs generated today are mainly of the ventricular and atrial type [10,25]. These cells are suitable for the study of the human myocardium, but they are not suitable for the study of cardiac pathologies related to the dysfunction of the sinoatrial node (SAN).

The SAN or sinus is the main cardiac pacemaker that controls heart rate. Pacemaker cells are unique in their ability to rhythmically self-depolarize, a characteristic known as automaticity [26]. Failure of SAN function due to congenital disease or aging leads to slow heart rate and inefficient blood flow, a condition treated by implanting an electronic pacemaker. The ability to produce pacemaker cells in vitro could offer an alternative biostimulator therapy in which the failing SAN is replaced by cell transplantation. Generating SAN-like cells from hiPSCs would facilitate the study of human SAN development and disease and provide a cellular source for the development of a biological pacemaker (Figure 1C). Biological stimulators represent a promising alternative to electronic stimulators, which have a number of disadvantages, including lack of hormonal responsiveness, risk of infections, limited battery life, and inability to adapt to changes in heart size in patients [27,28,29].

Given recent technologies, it is now possible to generate SAN-like cardiomyocytes from the patient [30,31,32]. This provides an exceptional opportunity to study the development and function of a human cardiac pacemaker, to model diseases that affect this cardiomyocyte subpopulation, and to design regenerative medicine for patients with SAN dysfunction. Recently, researchers have explored various methods for generating biological pacemakers, including using viral gene therapy to overexpress or suppress ionic currents, and differentiating hiPSCs as alternative treatments for cardiac conduction system disorders [28,33]. Here, we review the benefits and limitations of current protocols for directly generating SAN-like cardiomyocytes from human embryonic stem cells (hESCs) and iPSCs without using viral gene therapy to alter ionic current expression or key proteins involved in pacemaking. Additionally, we discuss therapeutic strategies involving the engraftment of SAN-like cardiomyocytes in animal models.

SAN-like cardiomyocytes derived from hiPSCs could shorten ex vivo production times and enable the development of off-the-shelf universal cell lines, making biological pacemakers more affordable and scalable for treating patients with SND and conduction disorders. Furthermore, using autologous or matched cells to minimize immune responses could enhance the activity of biological pacemakers while reducing risks of arrhythmogenicity and tumorigenicity [28].

## 2. Pacemaker-like Differentiation Protocols

Primary isolated cardiomyocytes from the human heart were cultured to investigate the underlying pathophysiological mechanisms related to human cardiac arrythmias [34,35]. Despite their physiological significance for studying specific cardiac features, isolated cardiomyocytes encounter various limitations such as ethical constraints, experimental and technical challenges in obtaining fresh cells, short-term survival in culture, electrophysiological abnormalities, and limited tissue availability for research [36,37]. The discovery of hESCs in 1998 and their derivation from the blastocyte inner cell mass revolutionized research by providing pluripotent cells capable of self-renewal and differentiation into any somatic cell type [38]. Subsequent differentiation of hESCs into cardiomyocytes (hESC-CMs) offered a human-based cell model in 2001, overcoming interspecies differences and enabling comprehensive cellular analysis [39]. hESC-CMs became instrumental in drug discovery, toxicity testing, and exploring early cardiac development and arrhythmogenic mechanisms, offering promising therapeutic avenues [40]. While ethical barriers limited hESC-CM research, the advent of hiPSCs addressed these issues, providing an unencumbered model for disease modeling and therapeutic exploration while preserving the hESCs characteristics [7,9,14]. In vitro, hiPSCs can be differentiated into cardiomyocytes using specific growth factors that mimic the natural signaling pathways of embryonic cardiomyogenesis. This process generates pacemaker-, ventricular-, or atrial-like cardiomyocytes, depending on the specific cytokine combinations, timing, and concentration. The fate of cardiac cells subtypes in hiPSCs imitates the controlled mechanisms seen in embryos [10]. Today, hiPSC-derived cardiomyocytes predominantly exhibit ventricular and atrial phenotypes [10], serving well for myocardial research but inadequately addressing SAN-related cardiac pathologies. Limited protocols exist for differentiating hESCS/hiPSCs into SAN cells, as outlined below. Cardiac differentiation depends on three key families of protein growth factors: Wnt proteins, fibroblast growth factors, and members of the transforming growth factor superfamily, such as BMPs, transforming growth factor β (TGF-β), and activin A [41,42,43]. Numerous protocols have achieved significant cardiomyocyte yields by modulating specific signaling pathways, particularly the WNT/β-catenin pathway, which is pivotal in cardiac differentiation [44]. The first biological-like pacemaker was developed in 2002 by inhibiting the inward-rectified potassium channels (Kcnj2), responsible for I_K1_ current and known for its substantial strong negative resting potential, thus suppressing the excitability [45]. In this study, they investigated the use of a viral vector for gene transfer to reprogram quiescent cardiac myocytes into functional pacemaker cells. This resulted in the generation of spontaneous and rhythmic electrical activity within the ventricle, both in vitro and in vivo [45,46]. Subsequently, several studies overexpressed other key pacemaker ion channels, such as HCN1-4, or pacemaker-related genes like *TBX18*, *TBX3*, *ISL1*, *SHOX2*, and *MYH6* in an attempt to develop biological pacemakers [47,48,49,50,51,52,53,54,55,56]. This led to a greater yield of cells with pacemaker-like characteristics, although it raised safety concerns due to the heightened risk of oncogenicity [30]. In this review, we focus on hESC and hiPSC directed-differentiation protocols. Advantages and inconveniences of each available protocol are summarized in Table 1.

### 2.1. hESC Pacemaker-like Differentiation Protocols

The differentiation of hESCs into SAN-like cardiomyocytes relied on the activation followed by the inhibition of the WNT/β-catenin pathway. In 2010, Zhu et al. offered the initial evidence that hESC differentiation into SAN-like cardiomyocytes was achievable. Their study demonstrated that inhibition of Neuregulin/ErbB signaling post mesoderm induction promoted the generation of a pacemaker-like population. While the specific phenotype of SAN or atrioventricular node (AVN) was not identified in the cells, the research highlighted the potential to generate pacemaker-like cells from hPSCs by modulating relevant signaling pathways [57].

In 2015, Christine Mummery’s group emphasized the critical role of precisely regulating the transition from cardiac progenitor cells (CPCs) to mature cardiomyocytes to enhance cardiac lineage specification. Specifically, they monitored the cardiac progression through the increasing expression of Pdgfr-α in CPCs and the progression of podoplanin (PDPN), which is predominantly expressed in the SAN [58]. Using hESCs engineered with Nkx2.5-eGFP and a doxycycline (dox)-inducible *MYC* transgene, they attempted to restrain Nkx2.5 enhancement by activating the *MYC* transgene with dox to yield SAN-like cardiomyocytes. Concurrently, they inhibited the NODAL pathway to reinforce Nkx2.5 downregulation. They found that maintaining these cells in the self-renewal state, combined with dox and NODAL inhibition, was insufficient for amplifying the population. To overcome this issue, they introduced mitogenic activators like insulin-like growth factor-1 (IGF-1) and a hedgehog agonist (Hh). Subsequently, this approach resulted in the generation of SAN-like cardiomyocytes with high expression levels of *TBX3*, *SHOX2,* and *HCN4* [58]. However, the overexpression of *MYC* may lead potentially to the development of pro-oncogenic cell types, limiting their application for generating biological pacemaker.

Li et al. investigated the effect of E2A ablation, a member of E proteins class I helix-loop-helix protein, on SAN-like cardiomyocyte differentiation using the hESC and CRSIPR/Cas9 technologies [59]. They suggested that the E2A protein interacts with Mesoderm Posterior BHLH Transcription Factor 1 (MESP1) transcription factor leading to the development of CPCs. Interestingly, reducing E2A levels during early cardiomyocyte differentiation indirectly increased the gene expression linked to cardiogenic mesoderm development. They found that the differentiated cells exhibited functional and morphological characteristics of SAN-like cardiomyocytes including higher *TBX3*, *TBX18*, and *SHOX2* mRNA level, higher beat rate, decreased contraction force, and smaller, rounder cardiomyocytes [59]. Unfortunately, the need of CRISPR/Cas9 genome editing to knock down E2A, its precise delivery, and potential off-target effects need to be addressed to ensure its suitability for direct therapeutic applications.

### 2.2. hiPSC Pacemaker-like Differentiation Protocols

Like hESCs, the differentiation of hiPSCs into SAN-like cardiomyocytes relied on the activation followed by the inhibition of the WNT/β-catenin pathway. The hiPSC differentiation into SAN-like cardiomyocytes protocol was developed by Gordon Keller’s group. In this study, they differentiated both a pre-modified eGFP-hESC line containing a marker for a heart development gene (*NKX2.5*) and a regular hiPSC line into embryoid body (EB)-like SAN cardiomyocytes [30]. These 3D cell clusters (three-dimensional cell aggregates) resemble early embryos after implantation [60]. Within EBs, cells undergo differentiation into derivatives representing the three primary germ layers: the ectoderm (outer layer), the mesoderm (middle layer), and the endoderm (inner layer). The obtained EBs expressed a high level of SAN-associated genes including *ISL1*, *SHOX2*, *KCNJ3*, *TBX3*, *TBX18*, and *HCN1-*4 as well as pacemaker-like AP characteristics such as slow maximum upstroke velocities, fast spontaneous firing rates, short action potential durations (APDs), and small action potential amplitudes (APAs). In line with a pacemaker phenotype, these SAN-like cardiomyocytes exhibited increased densities of both the ‘funny’ (pacemaker, I_f_) and the acetylcholine-activated potassium currents (I_KACh_) [30]. It is noteworthy that differentiating stem cells into embryoid bodies often results in a low yield of cardiomyocytes [61]. This makes it challenging for direct therapeutic applications, as these therapies require the transplantation of millions of cells. Recently, it has been suggested that using a bioreactor-based cardiac differentiation protocol could address this issue by producing higher-purity ventricular hiPSC-CMs with better post-thaw viability, greater reproducibility, and maturity compared to monolayer-derived CMs. This technique could potentially be adapted for differentiating SAN-like cardiomyocytes [62].

Subsequently, in 2020, Liu et al. adjusted Gordon Keller’s proposed protocol by differentiating hiPSCs into SAN-like cardiomyocytes using a 2D-based monolayer technique [31]. Two-dimensional cardiac monolayers are flat layers of cells, unlike 3D EBs, and are characterized by the absence of the endoderm and ectoderm, which typically surround the mesoderm layer [63]. The 2D-monolayer method is recognized for yielding a greater number of cardiomyocytes but with a lower degree of maturation compared to the 3D EB clusters [64,65]. In the proposed protocol, Liu et al. suppressed the fibroblast growth factor (FGF) signaling pathway that is crucial for ventricular development, used retinoic acid (RA) inhibitor, BMS, to prevent atrial, endocardial, and vessel specification, and employed a low concentration of BMP4 to promote SAN-like cardiomyocyte differentiation. Consequently, cells subjected to this approach exhibited increased expression levels of *SHOX2*, *TBX3*, *TBX18*, and *HCN4* [31]. However, the reproducibility of this protocol is limited as the data presented are based on observations from a single hiPSC cell line. Given the known variations in hiPSC characteristics among donors, incorporating additional cell lines derived from different donors in future experiments would be beneficial for evaluating the potential variability in this protocol’s outcome.

In 2022, the same group used a simplified protocol where they administered only BMS on day 5 of differentiation. This approach resulted in an increased level of SAN-like cardiomyocytes, which exhibited higher gene expression levels of *TBX3*, *TBX18*, *SHOX2*, *CX30.2*, *ISL1*, and *HCN4* [66]. Nevertheless, this protocol lacks a comprehensive molecular and functional characterization, including the electrophysiological properties of pacemaker-like currents.

Another simplified protocol emerged in 2020 by Deborah Lieu’s group. They suggested that NODAL inhibitor SB431542, when introduced at the stage of cardiac mesoderm differentiation, resulted in the downregulation of PITX2c, a transcription factor known for its role in suppressing the SAN formation in the left atrium during cardiac development [32]. Immunostainings of the differentiated cells showed increased protein expression levels of TBX3, TBX18, SHOX2, and ISL1, along with an elevated *HCN4* mRNA level. These SAN-like cardiomyocytes exhibited pacemaker-like electrophysiological characteristics including a more depolarized maximum diastolic potential (MDP), reduced APA, slower maximum rate of rise, and decreased cell capacitance indicative of smaller cell size [32]. Nonetheless, a major limitation of this protocol is the focus on a single signaling pathway. The hiPSCs cardiomyocytes differentiation mimics fetal heart development, which is a very complex process that is characterized by a highly coordinated spatiotemporal sequence in which combining different signaling pathways modulators is crucial. The final outcome depends on several factors, including the concentration of these signaling molecules and the exact time they are introduced during the differentiation window.

The recent paper by Giannetti and collaborators in 2021 introduces perspectives on the physiological characteristics of I_f_ current in hiPSC-derived pacemaker cardiomyocytes during different stages of maturation (days 15, 30, and 60). The authors elucidate changes in the voltage dependence and activation kinetics of I_f_, a crucial component in the cardiac action potential’s (APs) diastolic depolarization phase, in SAN-like cardiomyocytes at varying maturation levels. Isoproterenol (ISO), a β-adrenergic agonist, induced a positive shift in I_f_’s activation curve, thereby accelerating the spontaneous spiking rate, whereas acetylcholine (ACh) or carbachol (CCh) stimulation of muscarinic receptors caused a leftward shift in the activation curve, reducing current amplitude and slowing down the spiking rate. However, the cardiac differentiation was conducted using the PSC Cardiomyocyte Differentiation Kit provided by ThermoFisher Scientific (Catalog number: A2921201, Waltham, MA, USA) which is known for its ability to produce cardiomyocytes with characteristics more similar to ventricular-like rather than SAN-like cardiomyocytes [67].

More recently, Wiesinger and collaborators in 2022 employed an adapted Protze-based protocol to map out the transcriptional modifications during SAN-like differentiation using both single-cell RNA sequencing (scRNA-seq) and a computational method known as Trajectory Inference (TI) [68]. These SAN-like cardiomyocytes displayed elevated expression levels of *ISL1*, *SHOX2*, *TBX3*, *TBX18*, *HCN1-4*, *CACNA1D*, *CACNA1G*, *KCNJ3*, and *TNNT2* resembling those of pacemaker-like phenotype. They found distinct clusters in the differentiated SAN-like cardiomyocytes, which resemble various subpopulations found in the in vivo SAN. Interestingly, these cells also shared a developmental origin with proepicardial cells, present nearby, which is consistent with in vivo observations, and highlighted that the WNT signaling pathway plays a crucial role in separating these two cell lineages during development. Additionally, they identified contributions of TGF-β and WNT signaling in the differentiation of transitional and head SAN-like cardiomyocytes subtypes, respectively [68]. The SAN exhibits morphological and electrophysiological subpopulations such as head, tail, and transitional cells. PCs in the SAN-head population are known to express the T-box transcription factors *TBX18* and *TBX3*, and they lack the expression of *NKX2.5*. The SAN-tail population expresses *TBX3* and *NKX2.5*, but they are devoid of *TBX18*. The transitional cells have intermediate properties of PCs and atrial myocardium [68,69]. Finally, these SAN-like cardiomyocytes exhibited pacemaker-like electrophysiological characteristics including shorter cycle length, a more depolarized MDP, lower APA, and slower upstroke velocity. Treatment with an I_f_ channel blocker, Ivabradine, resulted in an increase in cycle length that remained unaffected in the ventricular-like cardiomyocytes, confirming the increased yield of pacemaker-like cardiomyocytes in the proposed protocol [68]. However, a limitation of this protocol is the lack of comprehensive functional characterization of the differentiated cells, particularly regarding their electrophysiological properties such as the characterization of pacemaker-like currents, as well as protein-level analysis.

A recent study has shed light on the role of cadherin-5 protein (CDH5 or VE-Cadherin) in the differentiation of SAN-like cardiomyocytes. CDH5 is a glycoprotein involved in cell adhesion typically found in vascular endothelial cells. Introducing CDH5 during cardiac differentiation has been shown to increase the proportion of SAN-like cardiomyocytes [70]. Interestingly, CDH5 appears to have a synergistic effect on WNT/β-catenin signaling, enhancing T-cell factor (TCF) transcription factor activity. In fact, TCF proteins are involved in modulating the expression of target genes in reaction to WNT signaling, especially in cell proliferation, differentiation, and developmental processes [25]. The differentiated SAN-like cardiomyocytes exhibited increased protein expression and mRNA levels of pacemaker-like markers including *TBX3*, *TBX18*, *SHOX2*, *ISL1*, *HCN1-4*, *KCNJ3*, and *GJC1-3* along with decreased ventricular-like cardiomyocyte markers such as *MYL2* and *NKX2.5*. The electrophysiological assessment of these SAN-like cardiomyocytes revealed an elevated beat rate and reduced values for MDP, APA, maximal upstroke velocity, and the AP Duration at 50% repolarization (APD_50_). This was accompanied with a higher I_f_ current density reflecting a more pacemaker-like phenotype. Treatment with iCRT14, a selective β-catenin inhibitor, prevented the increase of pacemaker-like markers, highlighting the pivotal role of CDH5 in SAN-like differentiation [70]. Nevertheless, one limitation of this protocol that should be taken into consideration is that CDH5 may also inhibit WNT/β-catenin signaling by sequestering β-catenin into the cadherin complex on the cell surface, thus preventing TCF activation.

Schweizer and collaborators in 2017 offered a transgene-free method of SAN-like differentiation by co-culturing hiPSCs with mouse visceral endoderm-like (END-2) cells until day 12 in serum-free conditions. Subsequently, beating 3D-clusters were isolated and transferred to dishes with fetal bovine serum (FBS)-enriched medium. They found a pacemaker-like gene-associated phenotype characterized by reduced expression levels of *TBX5* and *NKX2.5* along with elevated expression levels of *SHOX2*, *TBX3*, *TBX18*, *SLC8A1*, *CACNA1D*, *CACNA1G*, and *HCN1-4* [71]. These differentiated SAN-like cardiomyocytes exhibited some hallmark of pacemaker cells including an increased firing rate in response to β-adrenergic stimulation; conversely, activation of muscarinic receptors by CCh and the application of Ivabradine resulted in a decreased beat rate. Interestingly, these SAN-like cardiomyocytes effectively paced neonatal rat ventricular myocytes in co-culture experiments. These differentiated cells exhibited spontaneous Ca^2+^ release, indicating a coupled-clock mechanism responsible for automaticity in pacemaker-like cardiomyocytes. Additionally, they displayed certain electrophysiological characteristics of a pacemaker-like phenotype, such as a more negative MDP, shorter APD, and faster maximal rate of depolarization [71]. However, co-culturing SAN-like cardiomyocytes with END-2 cells carries a significant xenogeneic risk, which raises concerns for potential medical applications.

To overcome the inconveniences related to co-culturing hiPSCs with END-2 cells, the Schweizer group developed three END-2 cell-independent protocols for obtaining SAN-like cardiomyocytes [72]. These new protocols combine activation/inactivation of WNT/β-catenin signaling, NODAL inactivation through SB431542, and RA administration at different time points or in different combinations. The fourth protocol, used as a reference, involves using the STEMdiff^TM^ Atrial Cardiomyocyte Differentiation Kit from STEMCELL Technologies (Catalog number: 100-0215, Vancouver, BC, Canada). Compared to the END-2 cell-dependent method, the STEMCELL protocol showed higher SAN-like marker expression with an upregulation of 12 out of 15 pacemaker-like genes tested such as *SHOX2*, *TBX3*, *TBX18*, and *HCN1-4*. The protocol using the SB431542 showed an increase in 10 out of 15 pacemaker-like genes. Immunostaining of STEMCELL protocol-generated cells for cardiac pacemaker-like markers revealed high protein expression levels of HCN1-4, Cx45, TBX3, TBX18, and SHOX2. These SAN-like cardiomyocytes generated with the STEMCELL protocol displayed an elevated firing rate upon β-adrenergic stimulation. Conversely, their beat rate decreased upon activation of muscarinic receptors by CCh and the application of Ivabradine [72]. Unfortunately, all these protocols lack of functional and electrophysiological analysis of these SAN-like cardiomyocytes.

Ren et al. developed a SAN-like differentiation strategy centered on manipulating the WNT pathway. They demonstrated that reactivating WNT/β-catenin signaling alone could reinforce the cellular characteristics of the pacemaker-like phenotype [73]. They found that the SAN-like cardiomyocytes displayed increased pacemaker-like markers including *ISL1*, *TBX18*, *TBX3*, *SHOX2*, *BMP4*, and *HCN4* as well as reduced *NKX2.5* expression levels. These SAN-like cardiomyocytes displayed electrophysiological-like characteristics of a pacemaker-like phenotype including a higher beat rate. Interestingly, these SAN-like cells were able to pace 3D bioprinted hiPSC-CMs in vitro. Additionally, they used zebrafish embryos for precise cardiac mesoderm fate mapping through photoconversion lineage tracing and identified Wnt5b as the key WNT ligand directing cardiac progenitor cells towards a SAN-like cardiomyocyte phenotype [73]. It is noteworthy that the canonical WNT signaling temporal activation during differentiation resulted not only in the production of SAN-like cardiomyocytes but also epicardial cells; therefore, the complex signal pathway implicated remains to be elucidated.

It is noteworthy that the majority of these studies focused on the characterization of the I_f_ current as a key marker of the pacemaker-like phenotype; however, the expression of the I_f_ current is also observed in ventricular-like cardiomyocytes, due to their immature state [8,74,75], indicating that more specific biophysical parameters including pacemaker currents such as the I_CaL_, I_CaT_, and I_KACh_ would provide more informative insights into the actual electrophysiological characteristics of these SAN-like cardiomyocytes.

**Table 1:** Summary of hiPSCs- and hESCs-derived SAN-like cardiomyocytes protocols.

Model Used	Key Signaling Pathway and Cytokine Used	Advantages	Limitations	References
hESCs, hiPSCs	BMP4, Activin A, VEGF, SCF, WNT activator CHIR99021, doxycycline MYC transgene, Activin/NODAL/TGF-β inhibitor SB431542, WNT inhibitor XAV939, bFGF, TGF-β1, IGF-1, hedgehog agonist (Hh), SAG.	- Through the manipulation of FGF and BMP signaling pathways, they managed to obtain SAN-like cardiomyocytes exhibiting the appropriate electrophysiological and contractile properties in 3D EBs approach. - MYC identification as a permissive factor in CPC self-renewal paves the way for using expandable PSC-derived CPCs for various applications related to modeling or regenerating the human heart. - The use of hiPSCs to replicate and confirm the obtained results.	- The overexpression of *MYC* may lead potentially to the development of pro-oncogenic cell types, limiting their application for generating biological pacemakers in vivo. - One major limitation is the complexity of this protocol.	[58]
hESCs	CHIR99021, WNT inhibitor IWR1, The use of CDM3 medium (RPMI1640; BSA; and ascorbic acid). E2A ablation.	- Increase of SAN-like cells from 5% to 40% that exhibited functional and morphological pacemaker cells characteristics. These observations were supported by an increase of key SAN transcription factors using RNA-seq and transcriptomic techniques.	- A major limitation of this study is the inability to isolate the funny current (I_f_) which reflects the mature phenotype of pacemaker-like cells and is crucial for generating the slow diastolic depolarization that leads to spontaneous action potentials. - The need of CRISPR/Cas9 genome editing to knock down E2A, its precise delivery, and potential off-target effects need to be addressed to ensure its suitability for direct therapeutic applications.	[59]
hESCs and hiPSCs	BMP4, Activin A, bFGF, WNT inhibitor IWP2, Retinoic acid (RA), SB431542, VEGF, and the FGF signaling inhibitor PD 173074.	- Producing around 55% of SAN-like cardiomyocytes that exhibited electrophysiological characteristics of pacemaker-like phenotype in a 3D EBs technique. - This protocol offers the possibility to produce SAN-like cardiomyocytes without necessitating genetic manipulation of hiPSCs, leading to potential development of clinically compliant biological pacemakers.	- Differentiating stem cells into EBs often results in a low yield of cardiomyocytes. This makes it challenging for direct therapeutic applications, as these therapies require the transplantation of millions of cells.	[30]
hiPSCs	CHIR99021, IWP2, BMP4, PD 173074 and RA inhibitor BMS.	- Using flow cytometry cell sorting, this protocol yields approximately 55% of SAN-like cardiomyocytes displaying gene-associated pacemaker-like phenotype in a 2D-based monolayer technique. - The differentiation using 2D-based monolayer technique is recognized for yielding a greater number of cardiomyocytes, which facilitates its direct therapeutic applications.	- The lack of functional characterization, particularly an electrophysiology study, demonstrating the pivotal pacemaker-like currents as well as the verification of the pacemaker-like markers at the protein levels. - The reproducibility of this protocol is limited as the data presented are based on observations from a single hiPSC cell line. - Given the known variations in hiPSC characteristics among donors, incorporating additional cell lines derived from different donors in future experiments would be beneficial for evaluating the potential variability in this protocol’s outcome.	[31]
hiPSCs	CHIR99021, IWP2, BMS.	- 26.67% of the differentiated cells exhibited some morphological, electrophysiological, and gene-expression features similar to pacemaker-like phenotype.	- This protocol lacks a comprehensive molecular and functional characterization, including the electrophysiological properties of pacemaker-like currents. - All their claims are based on a single hiPSC cell line. - There is a lack of in vivo characterization of these SAN-like cardiomyocytes.	[66]
hiPSCs	CHIR99021, IWR1, SB431542.	- The simplest protocol that exists so far. - The use of two hiPSC cell lines to overcome any potential variability. - Both hiPSC cell lines were able to differentiate into SAN-like cardiomyocytes with differentiation rates of 22% and 32%, respectively, that exhibited some morphological and electrophysiological characteristics of pacemaker-like phenotype.	- One major limitation of this protocol is the focus on a single signaling pathway. - The hiPSC cardiomyocytes differentiation mimics fetal heart development, which is a very complex process that is characterized by a highly coordinated spatiotemporal sequence in which combining different signaling pathway modulators is crucial. The final outcome depends on several factors, including the concentration of these signaling molecules and the exact time they are introduced during the differentiation window. - Another limitation of this protocol is the lack of characterization of some pacemaker-like currents such as the I_CaL_, I_CaT_, and I_KACh_.	[32]
hiPSCs	BMP4, Activin A, CHIR99021, XAV939, RA, ALK5 inhibitor SB431542, PD173074.	- This protocol yields approximately 54% of SAN-like cardiomyocytes displaying electrophysiological and gene-associated characteristics of pacemaker-like phenotype using a 2D-monolayer approach. - The scRNA-seq allowed the classification of these differentiated SAN subpopulation phenotypes: 36% SAN-head, 19% SAN-tail, and 17% transitional. These insights shed new light on the molecular mechanisms governing human pacemaker cell development, paving the way for enhanced disease modeling in vitro and informing strategies for SAN regeneration in cell-based therapies.	- A limitation of this protocol is the lack of comprehensive functional characterization of the differentiated cells, particularly regarding their electrophysiological properties such as the characterization of pacemaker-like currents, as well as protein-level analysis. - Testing these differentiated SAN-like cardiomyocytes in vivo would have been informative to assess their viability as a potential biological pacemaker cell-based therapy.	[68]
hiPSCs	CHIR99021, IWR1, cadherin-5 (CDH5).	- This protocol results in around 23% of SAN-like cardiomyocytes exhibiting electrophysiological and gene-associated pacemaker-like characteristics using a 2D-monolayer technique. - Employing two different hiPSC cell lines helps mitigate potential variability in experimental outcomes. - This protocol demonstrates superior attributes in terms of simplicity, controllability, and cost-effectiveness.	- CDH5 may also inhibit WNT/β-catenin signaling by sequestering β-catenin into the cadherin complex on the cell surface, thus preventing TCF activation. - Another limitation of this protocol is the lack of characterization of pacemaker-like currents such as the I_CaL_, I_CaT_, and I_KACh_. - The SAN-like cardiomyocyte pacing potential has not been validated in vivo using animal models.	[70]
hiPSCs	p38 mitogen-activated protein kinase (MAPK) inhibitor SB 203580, ascorbic acid, Rho-associated protein kinases (ROCK) inhibitor Y-27632 dihydrochlorid, mouse visceral endoderm-like (END-2) cells.	- Through AP assessment, this method achieves approximately 63.4% of SAN-like cardiomyocytes demonstrating electrophysiological and gene-associated characteristics of pacemaker-like features when employing a 3D-cluster approach. - The differentiated SAN-like cardiomyocytes were able to induce pacing in neonatal rat ventricular myocytes during co-culture experiments. - Using three distinct hiPSC cell lines helps reduce the potential variability in these experimental results.	- Co-culturing SAN-like cardiomyocytes with END-2 cells carries a significant xenogeneic risk, which raises concerns for potential medical applications. - The interaction mechanisms between END-2 cells and hiPSCs remain unclear, making it challenging to predict how alterations in END-2 cell culture could impact hiPSC differentiation. - Co-culture and serum-dependent cardiac lineage specification need to be more elucidated. - The lack of current-like pacemaker phenotype characterization.	[71]
hiPSCs	CHIR99021, IWR1, RA, SB431542.	- Testing four protocols for SAN-like cardiomyocytes differentiation that exhibited some gene-associated pacemaker-like markers. - These four protocols are simple and easy.	- All these claims are based only on the pacemaker-like gene expression which does not reflect the biophysical outcomes of these protocols. - Lack of functional and electrophysiological analysis of these SAN-like cardiomyocytes.	[72]
hiPSCs	CHIR99021, IWP2.	- This protocol resulted in around 50% of SAN-like cardiomyocytes exhibiting electrophysiological and gene-associated pacemaker-like characteristics using a 2D-monolayer technique. - The SAN-like cardiomyocytes were able to pace 3D bioprinted hiPSC-CMs offering potential avenues for biological pacemaker therapy.	- Similar to the other protocols, this one also lacks electrophysiological current assessment. - In vivo pacing capability of these SAN-like cardiomyocytes has not been tested. - Canonical WNT signaling temporal activation during differentiation resulted not only in the production of SAN-like cardiomyocytes but also epicardial cells; therefore, the complex signal pathway remains to be elucidated.	[73]

hESC: human embryonic stem cells, EB: embryoid bodies, hiPSC: human induced pluripotent stem cells, SAN: sinoatrial node, CPC: cardiac progenitor cells, AP: action potential.

## 3. Engraftment of SAN-like Cardiomyocytes in Animal Models as a Potential Biological Pacemaker

Animal models such as guinea pigs, dogs, and pigs have been used as intact animal screens in previous studies [76]. Testing the differentiated SAN-like cardiomyocytes in vivo is crucial for evaluating potential therapeutic applications. Various animal models, both large and small, are vital for studying cardiovascular diseases, as they help assess disease mechanisms, diagnostics, and treatments. Large animals, in particular, offer valuable insights by closely mimicking human disease characteristics due to their close anatomical and physiological resemblance to humans also allowing for translating bench findings into effective treatments. Despite the challenges associated with large animal models such as the scarcity of transgenic models, advancements in gene editing are improving the development of tailored large animal models for human disease research [5,77,78]. The first biological pacemaker proof of concept was proposed by Eduardo Marban’s group by downregulating the inward rectifier current channel, I_K1_, using a viral vector for gene transfer to reprogram quiescent cardiac myocytes into functional pacemaker cells [45]. This construct, along with green fluorescent protein (GFP), was delivered using an adenoviral vector and administered into the left ventricular cavity of guinea pigs. Within 3 to 4 days, 80% suppression of I_K1_ was observed, along with the electrocardiographic manifestation of idioventricular rhythms in the treated guinea pigs’ hearts. Additionally, AP recordings from myocytes isolated from these hearts showed phase 4 depolarization and rapid automatic rates [45]. Reducing repolarizing current as described leads to a 15% prolongation of repolarization [79]. This presents concerns that this may lead to a potential proarrhythmic effect due to both increased dispersion of repolarization and the possibility of early after-depolarizations (EADs). The pacemaker’s reliance on several inward currents in the absence of a strong I_K1_ current creates uncertainty. This variability in how the heart responds electrically can cause unpredictable and potentially different rhythms between individuals and even within different regions of the heart [76]. Additionally, the safety implications of using viral injection raise apprehensions about its therapeutic use. hESCs along with hiPSCs hold immense promise for developing new cell-based therapies. The engraftment of SAN-like cardiomyocytes derived from hESCs and/or hiPSCs, as a potential biological pacemaker in vivo, is detailed in Table 2.

It was demonstrated that hESC-CMs, when injected into guinea pigs and swine models with AV block, successfully integrated into and paced these animals’ hearts [80,81]. Despite these promising results, significant challenges remain before this approach can advance to clinical applications. These include producing substantial quantities of purified cardiomyocyte cultures, preventing immune rejection, and proving that the grafts not only survive long term but also enhance myocardial function in diseased hearts. A significant concern is the potential formation of hESC-related tumors like teratomas. Ensuring grafts are free of undifferentiated hESCs is crucial to minimizing this risk. Finally, it is conceivable that early stage hESC-derived cardiomyocytes might mature over time into functional ventricular-like cardiomyocytes, potentially diminishing their ability for spontaneous pacemaking, therefore, making it challenging for a long-term application [82].

Protze et al. injected both SAN-like cardiomyocytes and ventricular-like cardiomyocytes near the apex of the rat’s LV myocardium to assess their pacemaking potential. They observed that six out of seven hearts that received SAN-like cardiomyocytes exhibited ventricular ectopic rhythm, whereas only one out of eight hearts that received ventricular-like cardiomyocytes displayed this escape rhythm. Optical mapping experiments revealed that the ectopic rhythm initiated at the septum is away from the region of ventricular-like cardiomyocytes cell transplantation. The SAN-like cardiomyocyte graft displayed positive staining for TBX3 but not for MLC2V or NKX2.5, while the ventricular-like cardiomyocyte graft exhibited the opposite pattern. Confocal imaging of anti-Cx43-stained sections revealed shared Cx43 channels between the engrafted cells and the rat cardiomyocytes, indicating electrical integration of the human cells with the host tissue [30].

Chauveau et al. have demonstrated the ability of hiPSC-derived EBs to integrate into the host myocardium and establish a functioning biological pacemaker in a canine atrioventricular (AV) block model for a 3-month follow-up [83]. They found that these EBs lost their pluripotency markers, acquired cardiac-specific markers, and exhibited I_f_-dependent automaticity. Epicardial pacing of the injection site revealed synchronized beats originating from that location as early as week 1 post-implantation. By week 4, 20% of beats were electronically paced, with 60% to 80% matching the originating site. Furthermore, the application of epinephrine elevated the rate of matching beats. Finally, the application of the vital dye Dil confirmed the persistent presence of injected cells at the administration site [83]. However, the escape times of the pace-mapped rhythms, ranging from 3.5 to 4.9 s, are inadequate. Moreover, in this study, the differentiation of hiPSCs was not guided by a specific protocol aimed at creating SAN-like cardiomyocytes. Instead, they employed EBs during the differentiation process, the spherical shape of which prevented cells not at or close to the surface from engaging with the surrounding myocardium. Consequently, the inward current crucial for phase 4 depolarization will be diluted by the substantial I_K1_ of neighboring ventricular myocytes. Therefore, it is crucial that the complete depolarizing current generated by the hiPSC-CMs be evenly distributed to the myocardium in a predictable manner [83].

**Table 2:** Summary of the engraftment of SAN-like cardiomyocytes, derived from hESCs and/or hiPSCs, as a potential biological pacemaker in vivo.

Animal Model Used	Injected Cells	Findings	References
Guinea Pig	hESC-derived CMs administered subepicardially using a 21-gauge needle into the LV anterior wall.	Optical mapping of the epicardial surface verified the effective propagation of membrane depolarization from the injection site to the neighboring myocardium.	[81]
Swine	hESC-CM cell clusters were injected via a left thoracotomy, targeting a site in the posterolateral wall of the LV.	The transplanted hESC-CMs successfully integrated in vivo and effectively paced the hearts of swine exhibiting complete atrioventricular block. This was confirmed through episodes of new ventricular ectopic rhythms observed through 3D-electrophysiological mapping and histopathological analysis. Interestingly, this new rhythm responded to adrenergic stimulation. These findings highlight the potential of hESC-CMs to function as a rate-responsive biological pacemaker and their promise for future myocardial regeneration approaches.	[80]
Rat	After performing a left thoracotomy, 0.5–2 million SAN-like cardiomyocytes were injected into the LV anterior wall close to the apex using a 28-gauge needle. Cyclosporine A (15 mg/kg/day) and methylprednisolone (2 mg/kg/day) were administered to the animals to prevent rejection of the human cell grafts by the immune system.	Following AV block induction, six out of seven hearts that underwent the SAN-like cardiomyocytes transplant showed ventricular ectopic beats. These ranged from isolated ectopic beats to sustained ectopic pacemaker activity lasting up to 60 s, with an average rate of 137 bpm. Optical mapping confirmed that these ectopic beats originated from the transplantation site at the heart’s apex. The human cell graft within the rat hearts was identified through immunostaining using an antibody that targets human cardiac troponin T (cTNT).	[30]
Canine	hiPSC-derived EBs were injected into the anterobasal region of the LV through an incision in the fifth left intercostal space. These injections were made approximately 3 to 4 mm deep into the epicardium using a 16-gauge needle, with a total volume of 0.4 to 0.7 mL of phosphate-buffered saline solution. It should be noted that a pacemaker lead (Dextrus model) was also inserted into the RV apex using a jugular venous approach.	The average and maximum biological pacemaker rates were recorded at 45 and 75 bpm, respectively. Histological examination using the vital dye Dil demonstrated the continued presence of injected cells at the administration site.	[83]

hESC-CMs: human embryonic stem cells-derived cardiomyocytes, hiPSC-derived EBs: human induced pluripotent stem cells-derived embryoid bodies, LV: left ventricle, SAN: sinoatrial node, AV: atrioventricular.

**Figure 2:**
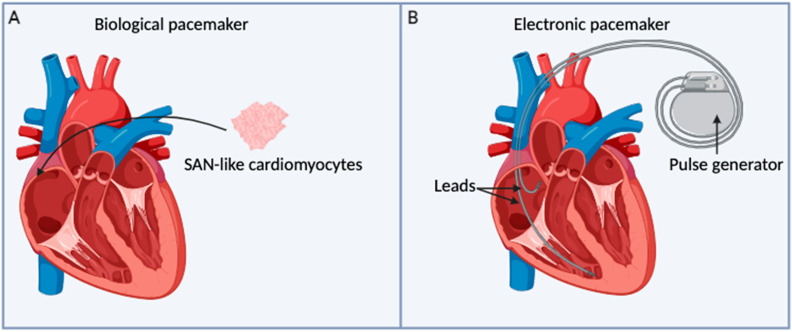
Illustration of a biological and an electronic pacemaker. (**A**) The SAN-like cardiomyocytes could be introduced into the patient’s heart, offering a promising cell-based therapy for developing a biological pacemaker. (**B**) A pulse generator is a compact metal device containing electronic circuitry, a battery, and a small computer, which together regulate the impulses sent to the heart. The leads are insulated wire connected to the pulse generator at one end with the other end positioned inside the heart’s chambers.

It is noteworthy that to achieve a successful biological pacemaker (Figure 2A), as defined by Komosa et al. [84], several crucial design criteria for reliable pacing must be met. The ideal biological pacemaker should perform the following:

- Generate spontaneous APs from a coupled membrane and calcium clock system, with clock protein expression at levels similar to the native pacemaker.
- Drive pacing over large regions of tissue by overcoming the source-sink ineffective pacing that fails to propagate properly.
- Provide reliable physiological pacing over extended periods with the ability to be self-sustaining and durable over the long term without requiring batteries, leads, electrodes or to be replaced or revised.
- Contain a similar level of heterogeneous pacemaker cell types as found in the SAN.
- Replicate the SAN tissue architecture, considering pacemaker and transitional cell patterning, extracellular matrix composition, and culture substrates.
- Demonstrate autonomic responsiveness, adjusting the pacing rate according to the body’s physiological needs.

Meeting these criteria is essential for clinical translation. Utilizing current knowledge of SAN structure, embryonic development, and addressing limitations of previous biological pacemaker developments will be crucial for success.

More research is needed to develop consistent guidelines that allow for a reliable correlation between the effectiveness of a biological pacemaker observed in vitro and in vivo. Despite, these challenges, biological pacemakers have demonstrated promise as proof of concept, and present a promising approach to replace electronic pacemakers that hold a number of disadvantages. Implanted electronic pacemakers encounter their own challenges explored below, and can play a direct role in the initiation or perpetuation of various types of arrhythmias, leading to a decline in the patient’s health. While new generation wireless pacemakers are available, the placement procedure remains invasive. These cardiac arrythmias related to electronic pacemaker dysfunction are discussed below. The development of biological pacemakers may encounter these challenges, which are crucial for the treatment and prevention of arrhythmias. A biological pacemaker has garnered significant interest due to its potential to mitigate device-related complications and ensure better physiological compatibility. This approach offers an appealing, device-free solution wherein heartbeats are produced by biological pacemaker cells, functioning similarly to those in natural human hearts [85].

## 4. Cardiac Arrythmias Related to Electronic Pacemaker Dysfunction

Pacemaker and/or conduction system disturbances may occur as a result of SAN-impaired impulse generation or blockage of impulse propagation at various points within the conduction system. Furthermore, conduction defects can occur due to other factors, including abnormal conduction pathways resulting from developmental anomalies [86]. Electronic pacemakers are surgically implanted in patients with cardiac rhythm abnormalities. Pacemakers are comprised two primary components: the pulse generator and the leads (Figure 2B). The pulse generator contains the battery and other electronic components that regulate the pacemaker’s modes of operation. The pacemaker leads are responsible for conducting the depolarizing potential to the myocardium, as well as sensing the intrinsic activity of the heart [87]. These pacing devices deliver an external electrical stimulus, initiating depolarization of myocardial cells, thereby maintaining the electrical excitability of heart tissue. This mechanism facilitates excitation-contraction coupling, leading to the contraction of myocardial tissue at a desired rate [86]. However, these devices can occasionally malfunction, leading to a range of symptoms that necessitate prompt evaluation and correction [88]. Electronic pacemaker dysfunction can be due either to sensing or pacing abnormalities. Sensing problems are characterized by under- or over-sensing. Under-sensing is characterized by the failure to detect intrinsic cardiac activity resulting in asynchronous pacing. It is caused by poor lead contact, programming problems, increased stimulation threshold, and new bundle branch block [89]. Over-sensing occurs when the pacemaker detects an electrical signal that it should not interpret as a relevant signal. This can lead to an inappropriate inhibition of the pacing stimulus, potentially resulting in life-threatening consequences [90]. Over-sensing arises when non-sinus electrical signals, such as large P- or T-waves, lead contact problems, and/or skeletal muscle activity are improperly recognized as native pacing activity and implanted pacemaker activity is halted [88,91].

**Figure 3:**
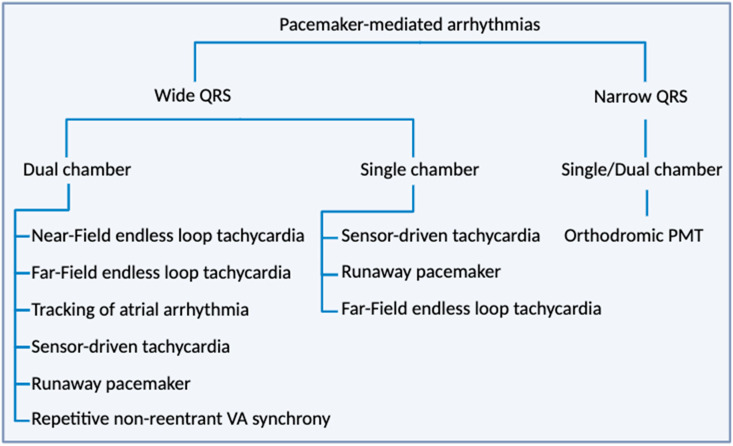
The diagram illustrates the various types of pacemaker-mediated arrhythmias observed in patients with both dual-chamber and single-chamber pacemakers. Adapted from [98].

Pacing abnormalities are characterized by failure to capture or output failure. Failure to capture occurs when stimulation does not lead to depolarization and signal propagation in the myocardium. This can be due to electrode displacement, electrolyte imbalance, myocardial infraction (MI), wire fracture, or exit block [92]. Output failure occurs when stimulation is abnormally generated resulting in a decrease or the complete absence of pacemaker function. It can be induced by wire fracture, over-sensing, interference, and lead displacement [88,93]. Several types of electronic pacemaker-associated cardiac arrythmias can occur and are detailed below. Figure 3 presents a diagram summarizing the various types of electronic pacemaker-mediated arrhythmias.

### 4.1. Antidromic Pacemaker-Mediated Reentrant Arrhythmias

The antidromic pacemaker-mediated reentrant arrythmias include the near- and far-field endless loop tachycardia. The endless loop tachycardia, also known as the pacemaker-mediated tachycardia (PMT) or pacemaker circus movement tachycardia, are triggered by ventricular premature beats (VPBs), atrial premature beats, and loss of atrial capture and involve a re-entry circuit through the AV node. In PMT, retrograde P-waves mimic atrial activity, causing a self-sustaining cycle of ventricular pacing and re-entry [94,95,96,97]. Tachycardia can be terminated by decelerating AV conduction with adenosine, magnet mode activation, carotid sinus massage, verapamil, or beta-blockers [95,98,99].

The far-field endless loop arrhythmia occurs when atrial sensing of ventricular activity initiates AV intervals, leading to AV dissociation and pacemaker syndrome. It can be prevented by prolonging the post-ventricular atrial refractory period (PVARP) [100,101]. The occurrence of these types of arrhythmias is less likely with biological pacemakers, as they are device-free solutions that function without leads and do not have a different refractory period.

### 4.2. Orthodromic Pacemaker-Mediated Tachycardia

Orthodromic pacemaker-mediated tachycardia, also known as reverse endless-loop tachycardia, is a rare arrhythmia where intrinsic AV conduction acts as the antegrade limb and the pacemaker as the retrograde limb. It can occur in dual-chamber pacemakers with synchronized atrial stimulation (SAS) or in single-chamber pacemakers due to far-field sensing of ventricular activation [100,101,102,103,104]. As mentioned above, this type of arrhythmias is less likely to occur with biological pacemakers, as they are device-free solutions that function without leads.

### 4.3. Repetitive Non-Reentrant VA Synchrony (RNRVAS)

Repetitive non-reentrant VA synchrony (RNRVAS) occurs in individuals with dual-chamber devices that feature AV sequential pacing and retrograde VA conduction. It resembles classic PMT and involves impaired atrial sensing, leading to a self-sustaining cycle of ventricular pacing [100,101,105,106]. It is noteworthy that rate-responsive modes and algorithms can increase the risk of this arrhythmia, which can be terminated by extending noncompetitive atrial pacing [105,106,107]. RNRVAS can disrupt efficient AV synchrony, causing symptoms like dyspnea, fatigue, and palpitations, and potentially exacerbating heart failure [108]. This type of arrhythmia is less likely to occur with biological pacemakers, as they do not have differing refractory periods and are likely to possess autonomic responsiveness, allowing the pacing rate to adjust according to the body’s physiological needs.

### 4.4. Tracking of Atrial Arrythmias or Myopotentials

The monitoring of atrial signals in atrial flutter, atrial tachycardia, or atrial fibrillation can lead to ventricular pacing reaching the maximum tracking rate, especially if the mode switch is inactive or under-sensing occurs [100,101]. Myopotential tracking is frequently observed in unipolar leads and is normally a result of monitoring the electrical activity of muscles [100,101]. A biological pacemaker, positioned directly on the heart, is potentially less prone to detecting extraneous electrical signals, and could be well positioned to address this issue.

### 4.5. Sensor-Driven Tachycardia

Sensor-driven tachycardia can occur in pacemakers with accelerometer-based sensors, often due to a low threshold or high slope setting, leading to elevated rates from minimal activity or non-physiological events [100,101]. It has been shown that replacing a generator with a smaller device programmed in a unipolar configuration has caused pectoral muscle stimulation and sensor-driven tachycardia, a complication not seen with bipolar pacing systems [109]. Biological pacemakers would be resistant to such disturbances and are likely to have autonomic responsiveness, enabling them to adjust the pacing rate based on the body’s physiological requirements.

### 4.6. Runaway Pacemaker

Runaway pacemaker is a malfunction, typically seen in older devices, where battery depletion or software errors cause the device to pace at dangerously high rates up to 200 bpm, potentially leading to ventricular fibrillation [100,101]. While rare in modern pacemakers, this condition requires urgent intervention, often necessitating device replacement; this is a surgical intervention and carries risks for the patient [110]. Biological pacemakers are a device-free solution that lack a battery and consequently will not undergo this phenomenon.

### 4.7. Pacemaker-Mediated Arrhythmias in Biventricular Pacing Systems

Pacemaker-mediated arrhythmias in biventricular pacing systems include cross-ventricular endless-loop tachycardia (interventricular PMT) and left ventricular (LV) upper rate interval (LVURI) lock-in. Cross-ventricular endless-loop tachycardia occurs when T-wave over-sensing causes LV pacing after a right ventricular (RV) event, which can be resolved by preventing T-wave over-sensing or deactivating LV triggering [111]. It may also arise from LV lead dislodgement into the coronary sinus, leading to left atrial capture and subsequent LV pacing [112,113]. Interventricular PMT can manifest in cases where a dual-chamber pacemaker has been adapted for biventricular pacing in patients with atrial fibrillation. This occurrence arises from T-wave over-sensing by the LV lead connected to the atrial port. Employing an appropriately programmed atrial sensing level can effectively mitigate T-wave over-sensing in this case [114,115].

LVURI lock-in arises when the LV’s upper rate limit has not been set higher that of the RV [116], potentially causing loss of resynchronization and decompensation of cardiac function, especially with the increasing use of devices with LV sensing parameters and LV T-wave protection options [117,118]. This type of arrhythmia is less probable with a biological pacemaker, as it operates without pre-set pacing rates, instead adapting autonomously to the body’s physiological demands.

### 4.8. Pacing Inducing Atrial or Ventricular Arrhythmias

These arrhythmias are infrequent, particularly in a pacemaker that functions normally and is not affected by secondary factors such as acute myocardial ischemia or metabolic abnormalities. Reports indicate that ventricular tachycardia can be induced by an abnormal circuit near the RV apex or by the fusion of pacemaker signals with numerous ventricular premature beats [119,120].

### 4.9. Pacemaker Lead Displacement Dysrhythmia

Pacemaker lead displacements refer to any shift in the pacemaker’s position, but only those causing pacing malfunctions are clinically significant. Displacements are classified as early (within six weeks of implantation) or late, and a dislodged lead can drift within the right ventricle, leading to ventricular ectopy or tachycardia. The diagnosis is confirmed through chest X-ray imaging [121]. This type of arrhythmias is less likely to occur with biological pacemakers, as they are device-free solutions that function without leads.

### 4.10. Pacemaker Syndrome

The pacemaker syndrome results from the incorrect timing of contractions between the atria and ventricles, leading to AV dyssynchrony and the absence of effective atrial contraction. It may occur when atrial contraction coincides with the closure of the AV valve or when atrial contraction occurs in close proximity to ventricular contraction, leading to back-pressure in the venous circulation systems and a reduction in the atrial contribution to ventricular output. Symptoms include palpitations, dizziness, fatigue, and pre-syncope. It is normally accompanied with systolic blood pressure reduction in exceeding 20 mmHg during the transition from the native rhythm to the paced rhythm [122].

### 4.11. Pacemaker Twiddler’s Syndrome

Pacemaker Twiddler’s syndrome is characterized by persistent malfunction of a pacemaker resulting from the patient’s manipulation of the pulse generator. The pacemaker undergoes rotation along its longitudinal axis, leading to displacement of pacing leads. It can lead to diaphragmatic or brachial plexus pacing depending on the degree of lead migration [123]. Biological pacemakers mitigate this concern by precluding patient manipulation of the pulse generator. Instead, they autonomously adjust pacing in response to physiological needs.

## 5. Conclusions and Perspectives

**Figure 4:**
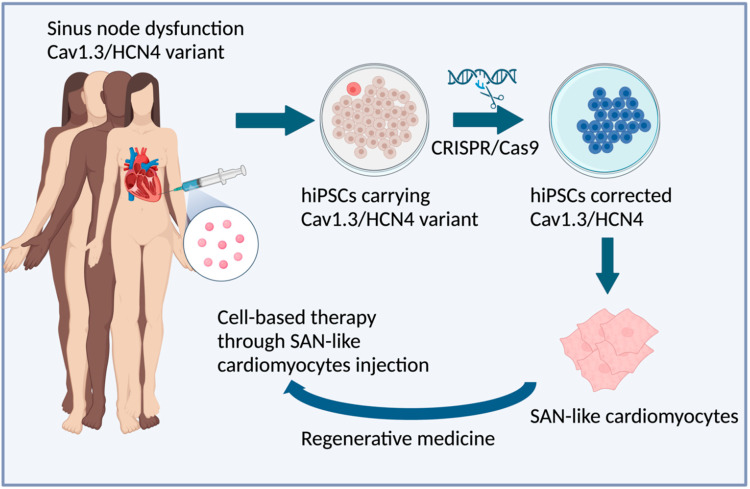
Illustration of the potential use of hiPSC-derived pacemaker cells. hiPSCs are generated from patients with genetic mutations associated with SAN dysfunction, such as Cav1.3 and HCN4. These mutations could then be corrected using CRISPR/Cas9 technology and differentiated into SAN-like cardiomyocytes. These SAN-like cardiomyocytes could be subsequently reintroduced into the patient’s heart. This approach holds promise as a potential cell-based therapy for a biological pacemaker.

The biological pacemakers developed over the last 20 years have demonstrated promise as proof of concept; however, they still present challenges for clinical implementation. While these developments have instilled some optimism, even the most encouraging functional outcomes achieved with gene-based constructs have not exhibited sufficient longevity to justify clinical translation. Furthermore, cell-based biological pacemakers, which have shown longer-term functionality, have been hindered by the requirement for immunosuppression and the associated risk of cell migration. While immunosuppression has historically posed a significant challenge in the utilization of hESC-derived pacemakers and xenogenic hiPSC-CM grafts in animal models, this concern would not apply to potential clinical applications of hiPSC-derived pacemakers where a patient’s own cells would be used to generate the graft. It would be intriguing to produce hiPSCs from patients with specific channelopathy mutations associated with SAN dysfunction, such as Cav1.3 and HCN4, which could then be corrected using CRISPR/Cas9 technology and subsequently reintroduced as SAN-like cardiomyocytes. This approach holds promise as a potential cell-based therapy for a biological pacemaker, as illustrated in Figure 4. This could provide a promising approach to replace electronic pacemakers that hold a number of disadvantages, including lack of hormonal responsiveness, risk of infections, limited battery life, and inability to adapt to changes in heart size in patients. From this perspective, the recent advancement in generating multi-chamber cardioids from hiPSC-CMs [124] represents a significant stride toward creating 3D models that could integrate SAN-like cardiomyocytes, derived from patients with SAN dysfunction, with atrial and ventricular chamber-like tissue. Such an approach would greatly enhance our comprehension of the mechanisms underlying SAN dysfunction. Furthermore, it would enable the testing of innovative pharmacologic or molecular strategies with potential clinical applicability. However, certain concerns need to be addressed and resolved before considering such an application. The time-dependent electrical changes in hiPSC/hESC-derived CMs, like changes in ion channel expression and action potential duration post-engraftment, may result in arrhythmias. Additionally, it is crucial to ascertain the ideal graft size for in vivo transplantation, assess the long-term survival and functional effectiveness of the transplanted cells, and evaluate their potential for adequate vascularization. Ultimately, the derivation and purification of a uniform and mature population of SAN-like cardiomyocytes will be essential. Single-cell RNA sequencing has been crucial in creating transcriptional roadmaps for human pacemaker cell differentiation. These profiles could guide the development of hiPSC-derived biological pacemakers, using developmental or lineage cues to achieve more mature and stable SAN-like characteristics. Biological pacemakers have shown promising developments in the past few years regarding SAN-specific differentiation protocols from both hESCs and hiPSCs. However, challenges persist including heterogenous cell phenotype and transient pacemaker automaticity expression. Therefore, more research is needed to develop a robust protocol that yields a high SAN-like cardiomyocyte population. Pre-clinical studies involving small and large animals will be monitored expectantly for further developments in this space.

## Abbreviations

AV	Atrioventricular
ACh	Acetylcholine
APs	Action potentials
APD	Action potential durations
APA	Action potential amplitudes
CVD	Cardiovascular disease
Ca^2+^	Calcium
CCh	Carbachol
GFP	Green fluorescent protein
hESCs	Human embryonic stem cells
hiPSCs	Human induced pluripotent stem cells
hiPSC-CMs	Human induced pluripotent stem cell-derived cardiomyocytes
hESC-CMs	Human embryonic stem cell-derived cardiomyocytes
ISO	Isoproterenol
LV	Left ventricle
MDP	Maximum diastolic potential
PMT	Pacemaker mediated tachycardia
PVARP	Post-ventricular atrial refractory period
RV	Right ventricle
SAN	Sinoatrial node
VPB	Ventricular premature beat
VA	Ventriculo-atrial
